# A Flagellin-Derived Toll-Like Receptor 5 Agonist Stimulates Cytotoxic Lymphocyte-Mediated Tumor Immunity

**DOI:** 10.1371/journal.pone.0085587

**Published:** 2014-01-14

**Authors:** Nicholas D. Leigh, Guanglin Bian, Xilai Ding, Hong Liu, Semra Aygun-Sunar, Lyudmila G. Burdelya, Andrei V. Gudkov, Xuefang Cao

**Affiliations:** 1 Department of Immunology, Roswell Park Cancer Institute, Buffalo, New York, United States of America; 2 Department of Medicine, Roswell Park Cancer Institute, Buffalo, New York, United States of America; 3 Department of Cell Stress Biology, Roswell Park Cancer Institute, Buffalo, New York, United States of America; McGill University Health Center, Canada

## Abstract

Toll-like receptor (TLR) mediated recognition of pathogen associated molecular patterns allows the immune system to rapidly respond to a pathogenic insult. The “danger context” elicited by TLR agonists allows an initially non-immunogenic antigen to become immunogenic. This ability to alter environment is highly relevant in tumor immunity, since it is inherently difficult for the immune system to recognize host-derived tumors as immunogenic. However, immune cells may have encountered certain TLR ligands associated with tumor development, yet the endogenous stimulation is typically not sufficient to induce spontaneous tumor rejection. Of special interest are TLR5 agonists, because there are no endogenous ligands that bind TLR5. CBLB502 is a pharmacologically optimized TLR5 agonist derived from *Salmonella enterica* flagellin. We examined the effect of CBLB502 on tumor immunity using two syngeneic lymphoma models, both of which do not express TLR5, and thus do not directly respond to CBLB502. Upon challenge with the T-cell lymphoma RMAS, CBLB502 treatment after tumor inoculation protects C57BL/6 mice from death caused by tumor growth. This protective effect is both natural killer (NK) cell- and perforin-dependent. In addition, CBLB502 stimulates clearance of the B-cell lymphoma A20 in BALB/c mice in a CD8^+^ T cell-dependent fashion. Analysis on the cellular level via ImageStream flow cytometry reveals that CD11b^+^ and CD11c^+^ cells, but neither NK nor T cells, directly respond to CBLB502 as determined by NFκB nuclear translocation. Our findings demonstrate that CBLB502 stimulates a robust antitumor response by directly activating TLR5-expressing accessory immune cells, which in turn activate cytotoxic lymphocytes.

## Introduction

Toll-like receptors (TLR) recognize highly conserved molecular patterns of bacteria, virus, and cells of host origin [1]. This feature allows TLR-expressing immune cells to respond rapidly to a pathological insult. In the presence of TLR agonists, antigen presenting cells (APCs) undergo a process of maturation characterized by up-regulation of costimulatory molecules, major histocompatibility complex (MHC) class II, and increased production of inflammatory cytokines. Mature APCs are then capable of providing a danger context, allowing the immune system to successfully respond to pathogenic antigens [2]. The danger context elicited by TLR agonists allows an initially non-immunogenic antigen to consequently become immunogenic. This ability to alter environment is highly relevant in tumor immunity, since tumors are from the host and it is inherently difficult for the immune system to recognize them as immunogenic. However, immune cells may have encountered certain TLR ligands associated with tumor development, yet this endogenous stimulus is typically not sufficient to induce spontaneous tumor rejection [3]. Previous reports suggest that quantity of ligand may be an issue, because various endogenous TLR agonists that target TLR3, TLR4 and TLR9, have shown various efficacies in boosting an antitumor response [4]–[6].

TLR receptors that only recognize exogenous ligands are an attractive alternative to TLR receptors recognizing endogenous ligands. Flagellin, the structural component of *Salmonella enterica* flagellum, is the only known ligand for TLR5 [7]. *In vitro* experiments using intestinal epithelial cells showed that TLR5 binding by flagellin initiates a signal transduction cascade leading to nuclear translocation of NFκB [8]. Because NFκB controls transcription of a variety of pro-inflammatory cytokines, it is not surprising that upon flagellin injection, there is an increase in circulating levels of TNF-α, IL-6, and IL-12 [8]. This response likely contributes to the ability of flagellin to promote both T cell and humoral responses [9]–[11]. Flagellin has been explored in mediating antitumor immunity. However, some tumor types may express TLR5 and the different timing of flagellin treatment may also cause varying effects, leading to conflicting results regarding whether flagellin actually promotes or suppresses tumor growth [11]–[13]. Meanwhile, a pharmacologically optimized TLR5 ligand has been developed from flagellin by replacing its hypervariable region with a short, flexible linker that connects two constant regions, which are essential and sufficient for TLR5 binding [14]. As a result the new product, CBLB502, elicits less of an antibody response to the agent itself when comparing serum levels of antibodies after either flagellin or CBLB502 administration [14]. Also, it shows twice the maximum tolerated dose as compared to flagellin, yet is as efficacious as flagellin in inducing NFκB nuclear translocation [14]. Due to significant reduction in immunogenicity and toxicity, CBLB502 has emerged as a more attractive TLR5 agonist.

Previous work from our lab has explored the ability of CBLB502 to promote CD8^+^ T cell responses following allogeneic bone marrow transplantation, a setting in which potent allogeneic antigen stimulation and pro-inflammatory cytokines are present [15]. In this study, we explored whether CBLB502 could provoke an effective danger environment and thus stimulate an antitumor immune response to syngeneic tumors, a setting in which allogeneic antigen stimulation and pro-inflammatory cytokines are not ubiquitous. To this end, we utilized two syngeneic lymphoma models that do not directly respond to CBLB502, since flagellin can directly interact with TLR5-expressing tumors and subsequently promote tumor growth or inhibit tumor growth depending on the tumor model [12], [13]. We have found that CBLB502 treatment after tumor inoculation stimulates a robust antitumor response that involves both innate and adaptive immune cells. Our findings demonstrate that CBLB502 stimulates tumor immunity via TLR5-expressing accessory immune cells which can then activate cytotoxic lymphocytes.

## Materials and Methods

### Animals

Mice were kept in SPF housing and maintained under the animal care guidelines at Roswell Park Cancer Institute (RPCI) under protocol ID numbers 1143 M and 1140 M. All experiments were approved by the animal studies committee at RPCI. WT C57BL/6 and WT BALB/c mice were purchased from the National Cancer Institute. Prf1^−/−^ and TLR5^−/−^ mice in the C57BL/6 background were obtained from the Jackson Laboratory and maintained as previously described [14], [16], [17].

### Reagents and Antibodies

Antibodies including anti-mouse CD3 (145-2C11), CD4 (RM4-5), CD8 (53-6.7), NK1.1 (PK136), CD11c (N418), CD11b (M1/70), CD80 (16-10A1), and CD86 (GL1) were purchased from eBioscience. Anti-asialo GM1 was obtained from Wako Chemical Industries and was given IV at a dose of 100 µg per mouse 3 days prior to tumor injection. Anti-CD8α depleting antibody from BioXcell was injected IP at 400 µg per mouse. CBLB502 was provided by Cleveland Biolabs. Trace amounts of endotoxin were removed from purified protein by detoxigel (Pierce) according to manufacturer’s protocol. CBLB502 was stored at −80°C in PBS and diluted in PBS or complete media directly before injections or use in vitro.

### Tumor Models and CBLB502 Treatment

RMAS T cell lymphoma, derived from the C57BL/6 background, was used in all studies with the C57BL/6 mice. 10,000 RMAS cells were injected intravenously into C57BL/6 recipients. 4 h after tumor inoculation, mice were treated with 100 µL CBLB502 (10 µg/mL) or 100 µL PBS. Mice were then treated every 48 hours through day 8. For co-culture experiments RMAS was transduced with a ΔU3 retroviral construct that drives expression of the fusion cDNA which encodes for both green fluorescence protein (GFP) and click beetle red (CBR) luciferase [17]. The BALB/c derived B cell lymphoma, A20, was similarly transduced and used both *in vivo* and *in vitro*. This line was used in all BALB/c IV inoculations at a dose of 1×10^6^ cells. Tumor burden was measured by bioluminescence imaging and expressed as photon flux (photons/sec) as previously described [17], [18]. Survival curves of tumor-challenged mice are representative of either the day in which mice were found dead, or were determined moribund by the department of lab animal resources and sacrificed. SCCVII, derived from C3H/HEN (H-2^k^) mice, is a squamous cell carcinoma tumor line that is known to express TLR5 [19].

### RT-PCR

For analysis of TLR5 mRNA expression, total RNA was extracted from A20, A20-luciferase, RMAS, RMAS-luciferase and SCCVII cell lines using TRIzol reagent according to manufacturer instructions (Invitrogen, Carlsbad, CA). cDNAs were synthesized and RT-PCR was performed as previously described [15].

### In vitro Co-cultures

C57BL/6 and BALB/c splenocytes were red cell lysed and resuspended in RPMI 1640 media supplemented with 10% heat-inactivated FBS, HEPES, non-essential amino acids, sodium pyruvate, L-glutamine, penicillin-streptomycin, and 2-mercaptoethanol. Splenocytes were mixed with tumor cells and added in 1 mL aliquots to 48 well plates. Luciferase-expressing RMAS and A20 tumor cells were used for co-cultures at the indicated ratios. At 0 hr 100 ng CBLB502 was added to treat the cells. After 72 hrs 20 µl luciferin (15 mg/ml) was added to the wells and IVIS imaging system (Xenogen) was used to determine tumor burden in each well.

### ImageStream Flow Cytometry

Splenocytes were treated with 10 ng/mL TNF-α, 100 ng/mL LPS, 100 ng/mL CBLB502 or PBS for 1 hr. Cells were stained for CD3, CD4, CD8, NK1.1, CD11b, CD11c and Live/dead fixable yellow dead cell stain (Invitrogen). Following incubation, cells were fixed and a previously described intracellular NFκB staining protocol was followed [20]. Briefly, cells were stained for NFκB p65-FITC and nuclear dye DAPI was added immediately before samples were acquired on ImageStreamX, with a minimum of 500 cells for every population being collected. Cells were gated as single cells that were in focus, live, DAPI positive and p65 positive as previously described [21]. Further gating was done to analyze CD3^+^CD4^+^, CD3^+^CD8^+^, CD3^−^NK1.1^+^, CD11b^+^CD11c^−^ and CD11b^+^ CD11c^+^ populations. The indicated populations were then analyzed for co-localization of p65-FITC and DAPI using the “Similarity” feature in the IDEAS® software package. “Similarity score” is a log-transformed Pearson’s correlation coefficient between the pixel values of two image pairs. In this case the image pairs are that of NFκB and DAPI and similarity score provides the degree of nuclear localization of p65 by measuring the pixel intensity correlation between these images. This feature was further described previously [20].

### Measurement of Cytokine Levels in Plasma

C57BL/6 mice were treated with 5 µg CBLB502 and blood was collected by cardiac puncture at various time points after treatment. Protein levels of cytokines were measured by Luminex multiplex assays, using a Luminex-100 Multiplex Bio-Assay Analyzer or Luminex-200 dual-laser flow analyzer (Luminex Corporation, Austin, TX) according to the manufacturer’s instructions.

## Results

**Figure 1:**
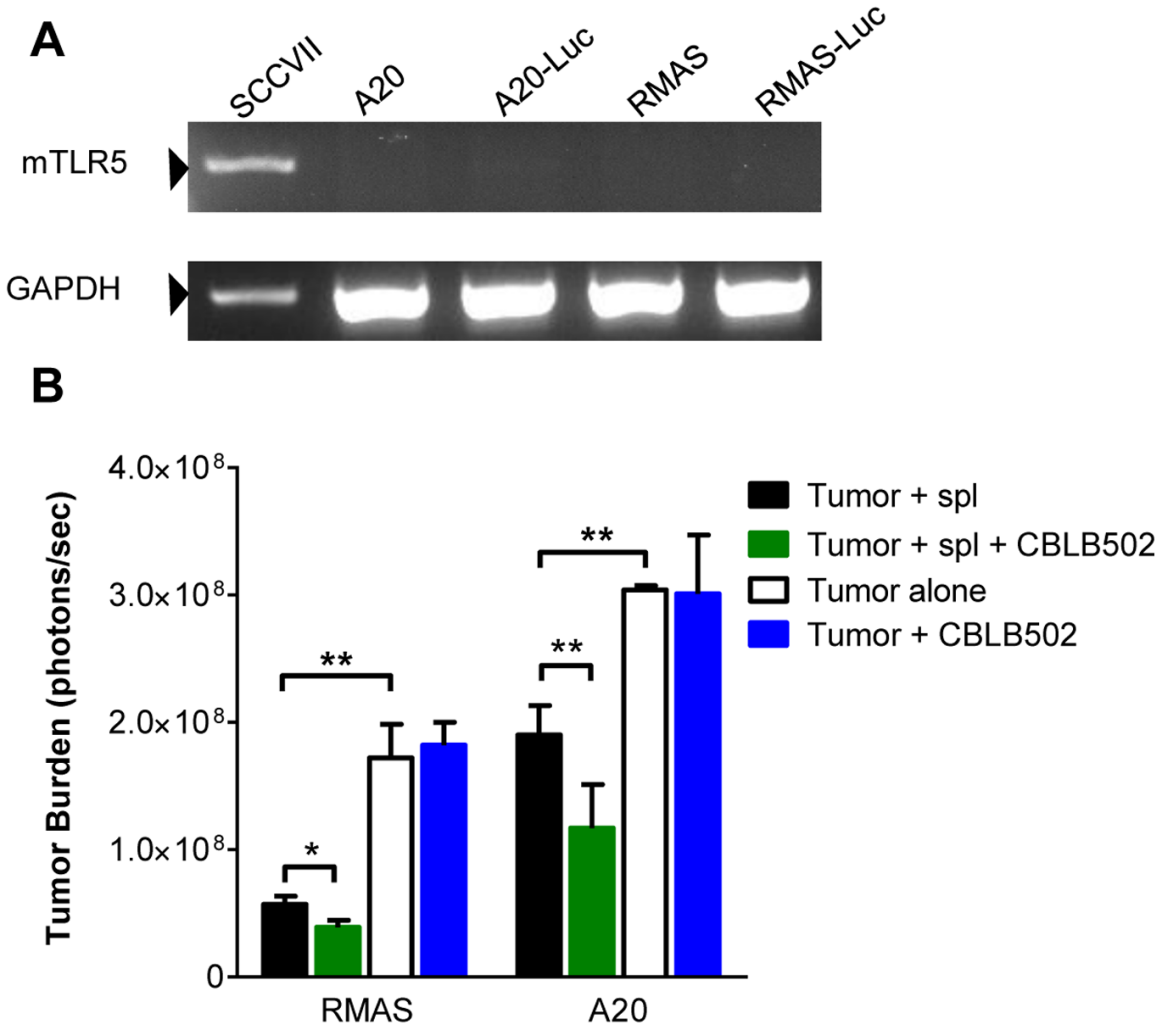
CBLB502 enhances the ability of splenocytes to control tumor growth *in vitro*. (A) SCCVII (a tumor line known to express TLR5 mRNA), A20 parental, luciferase-expressing, and RMAS parental and luciferase-expressing tumor cells were analyzed for TLR5 mRNA expression via RT-PCR. GAPDH was used as an internal loading control. (B) 2000 luciferase-expressing RMAS or A20 cells were cultured alone in 1 ml of complete media or co-cultured with 6×10^6^ C57BL/6 or BALB/c splenocytes, respectively. At 0 hr, the cells were treated with 10 µl (10 µg/mL) CBLB502 or media diluent. Tumor burden was measured by bioluminescence imaging after 72 hrs. Two tailed t-tests were used to determine significance (*P<0.05, **P<0.01). Shown are representative data from three independent experiments with similar results.

Previous studies have reported that TLR5 activation can promote or inhibit tumor growth dependent on the tumor model [12], [13]. To rule out this compounding factor and help establish a role for a host-derived immune response, we determined TLR5 mRNA expression in the tumor lines we use throughout this study. Both RMAS and A20 parental and luciferase-expressing lines had no detectable TLR5 mRNA (Fig. 1A). We hypothesized that CBLB502 would have the ability to activate immune cells, thereby controlling tumor growth. To confirm that CBLB502 did not directly affect tumor growth, we used CBLB502 to treat the tumor cell cultures; this yielded no change in tumor burden after 72 h treatment (Fig. 1B). These data rule out a direct effect of CBLB502 altering tumor growth and suggest that any change in tumor growth *in vivo* can be attributed to immune cells.

First, to test this hypothesis *in vitro*, splenocytes were harvested from naïve C57BL/6 mice and co-cultured with the luciferase-expressing RMAS tumor cells. Compared to the control wells cultured with tumor cells only, splenocytes were able to suppress tumor growth in the co-culture wells. As expected, CBLB502 significantly enhanced the ability of splenocytes to control tumor growth (Fig. 1B). To test the *in vitro* antitumor activity with A20 tumor, these experiments were repeated with BALB/c mice, revealing that splenocytes had the ability to suppress A20 tumor growth, and that CBLB502 treatment further increased their tumor control activity (Fig. 1B). These results establish that CBLB502 had a boosting effect on immune cell function *in vitro.*

### CBLB502 Stimulates a NK Cell- and Perforin-dependent Antitumor Immune Response

**Figure 2:**
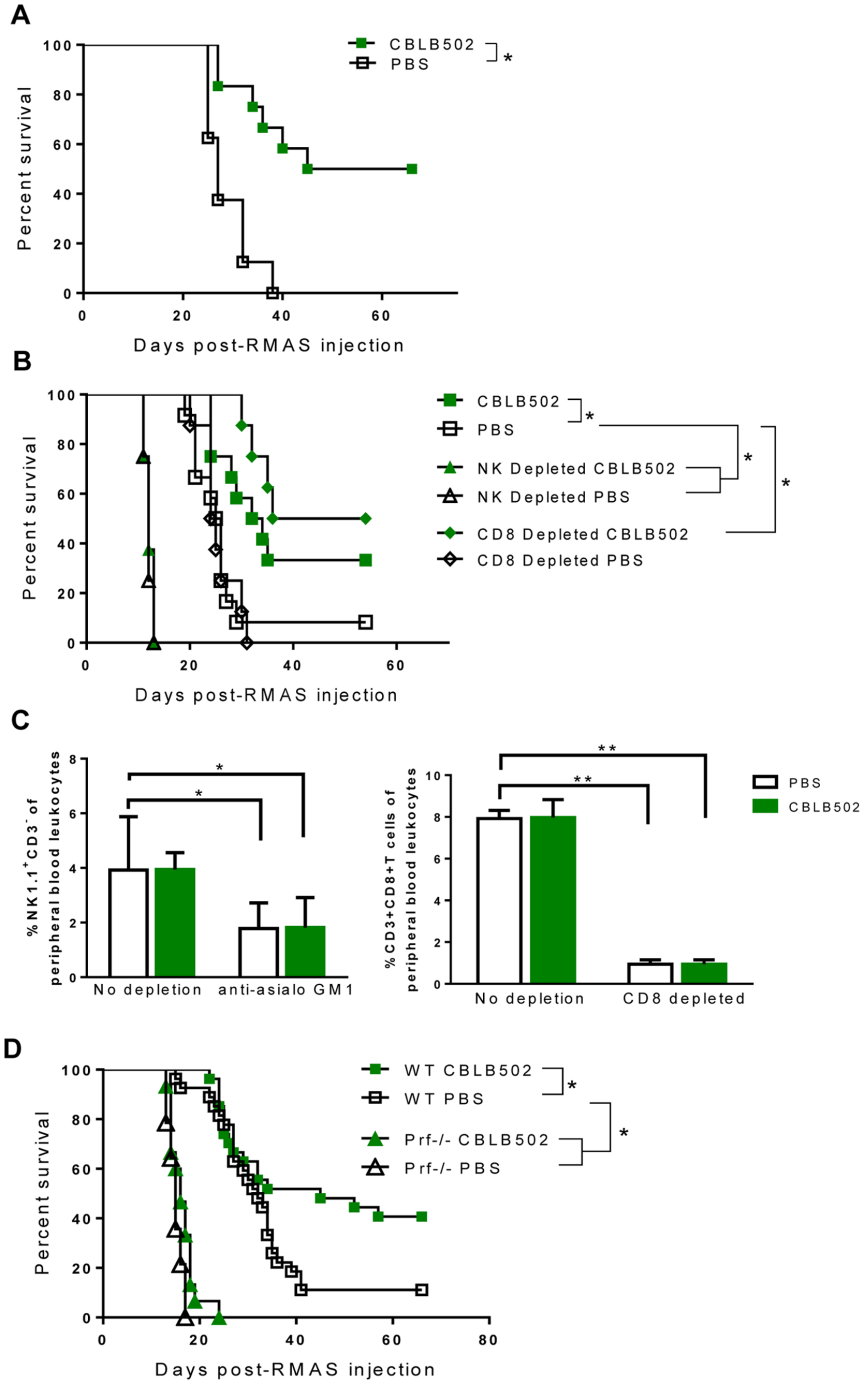
CBLB502 stimulates NK cell- and perforin-dependent tumor immunity. (A) Kaplan-Meier survival curve of WT C57BL/6 mice injected intravenously (IV) RMAS cells. Mice were treated with subcutaneous (SC) injections of either CBLB502 or PBS as described in Materials and Methods (PBS n = 8, CBLB502 n = 12). (B) Kaplan-Meier survival curve of WT C57BL/6 mice that were non-depleted or given either anti-CD8α antibody or anti-asialo GM1 antibody three days prior to IV injection with RMAS cells. PBS (n = 8), non-depleted CBLB502 (n = 12), NK depleted PBS (n = 8), NK depleted CBLB502 (n = 8), CD8 depleted PBS (n = 8), CD8 depleted CBLB502 (n = 8). (C) Peripheral blood was collected via eye bleeding 2 weeks after depletion with anti-asialo GM1 or anti-CD8α and the percentage of NK1.1^+^CD3^−^ and CD3^+^CD8^+^ T cells, respectively, were analyzed (n = 8 mice/group). (D) Kaplan-Meier survival curve of WT CBLB502 (n = 27), WT PBS (n = 27), Prf1^−/−^ CBLB502 (n = 15), Prf1^−/−^ PBS (n = 14). All asterisk (*) represent statistical significance as determined by Log-rank (Mantel-Cox) test versus non-depleted PBS control group (*P<0.05). All data shown are representative of one of at least three individual experiments, or data combined from multiple experiments.

Since CBLB502 treatment *in vitro* promoted splenocyte mediated control of tumor growth, we sought to determine whether CBLB502 could increase survival of C57BL/6 mice challenged with syngeneic RMAS lymphoma. Mice were intravenously inoculated with RMAS and treated with CBLB502 as described in the Materials and Methods. Endotoxin was removed from all CBLB502 used throughout to ensure specificity to TLR5. We found that CBLB502-treated mice had significantly improved survival with clearance of tumors, while no PBS-treated mice survived (Fig. 2A). RMAS cells have extremely low major histocompatibility complex (MHC) class I expression [17]. Since MHC class I inhibits NK cells ability to kill targets, RMAS cells are susceptible to NK cell-mediated killing [22]. To test whether NK cells were involved in CBLB502 stimulated tumor control, we used anti-asialo GM1 antibody to deplete NK cells before we challenged the mice with RMAS and treated with CBLB502. Anti-asialo GM1 is expressed by cell types including NK cells, basophils and T cells [23]. However, previous work has shown that upon depletion with NK1.1 antibody, that NK cells are required for controlling RMAS growth, while CD3, CD4 and CD8 antibody depletions had no effect on tumor growth versus non-depleted controls [24]. As seen in Figure 2B, the ability of CBLB502 to promote host survival is completely abrogated in anti-asialo GM1 treated mice, with the PBS- and CBLB502-treated mice having nearly identical survival curves. The extend of these depletions was checked 2 weeks after initial depletion, and NK cells in both anti-asialo GM1 PBS and CBLB502 were still significantly lower than non-depleted PBS controls (Fig. 2C). This result is consistent with previous work showing that mice genetically lacking NK cells have markedly diminished ability to clear MHC I deficient tumors [25].

However, RMAS is not completely MHC class I deficient, making a contribution from CD8^+^ T cells in tumor clearance a possibility. To examine the contribution of CD8^+^ T cells in RMAS clearance, we depleted CD8^+^ T cells from the mice 72 h prior to challenge with RMAS. CD8^+^ T cells were still markedly reduced 2 weeks following the depletion (Fig. 2C). We observed similar results to that of non-depleted controls, with CBLB502 rescuing a significant portion of CD8^+^ T cell-depleted mice (Fig. 2B). These data suggest that CD8^+^ T cells do not contribute to the control of RMAS tumor and CBLB502 is promoting NK cell-mediated tumor clearance.

A major mechanism by which NK cells mediate tumor killing is the perforin/granzyme pathway [26]. To test the involvement of this pathway in CBLB502-stimulated tumor clearance, we challenged perforin-deficient (*Prf1−/−*) mice with RMAS cells. The CBLB502 effect was completely abrogated, suggesting that CBLB502 treatment was stimulating tumor clearance via a perforin-dependent mechanism (Fig. 2D). Together, these data show that CBLB502 administered after tumor challenge has the ability to significantly improve host survival by promoting perforin-dependent and NK cell-mediated RMAS tumor clearance.

### CBLB502 Stimulates a CD8^+^ T cell-dependent Antitumor Immune Response

**Figure 3:**
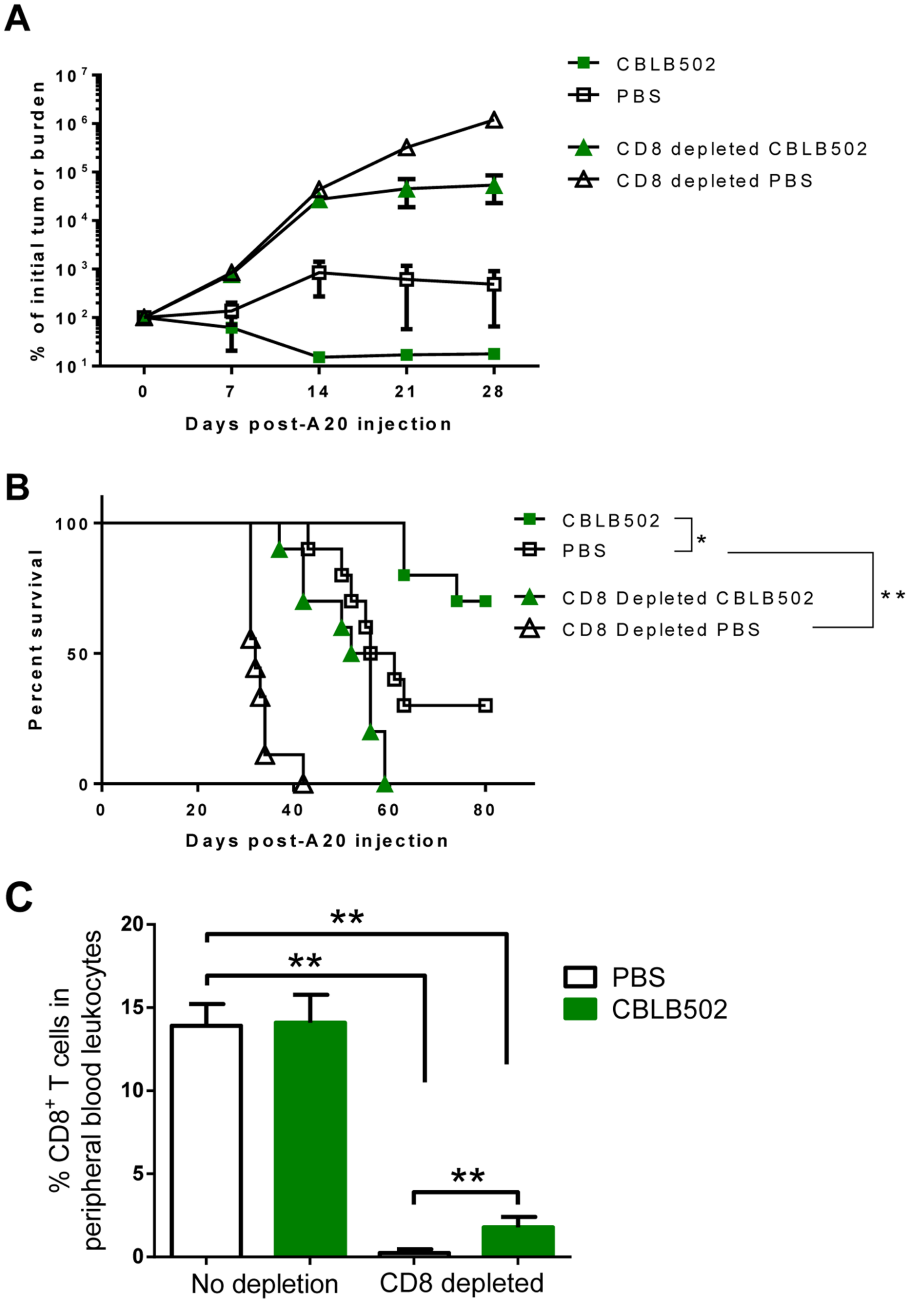
CBLB502 treatment stimulates CD8^+^ T cell-dependent tumor immunity. (A) Tumor burden measured by bioluminescence imaging in BALB/c mice that were either non-depleted or given anti-CD8α antibody 3 days prior to IV inoculation with A20-luciferase expressing cells. Four different groups CBLB502 (n = 8), PBS (n = 8), CD8 depletion CBLB502 (n = 7) and CD8 depletion PBS (n = 8) were treated with PBS or CBLB502. (B) Kaplan-Meier survival curve of BALB/c mice from the experiment described in panel A. Data are combined from 2 of 3 individual experiments. (C) To confirm depletions of CD8^+^ T cells, peripheral blood was harvested via eye bleed day 16 after depletion and the percentage of CD3^+^CD8^+^ T cells in peripheral blood leukocytes was analyzed (n = 3–4 mice/group). Two tailed t-tests were performed to evaluate significance (**P<0.01). Shown as mean ± SD. Statistical analysis of survival was performed by Log-rank (Mantel-Cox) test (*P<0.05) with all groups being compared to PBS-treated non-depleted cohort.

To confirm this was not a tumor line- or mouse strain-specific anomaly, we challenged BALB/c mice with the B cell lymphoma A20. Mice were inoculated intravenously with luciferase-expressing A20 cells and treated with CBLB502. Unlike RMAS, A20 has both MHC class I and class II, making them likely candidates to be targeted by the adaptive immune response [27]. This model allowed us to determine whether CBLB502 could also stimulate an adaptive immune response to A20 as opposed to the innate response seen in the RMAS model. To test this hypothesis, CD8^+^ T cells were depleted 3 days before A20 tumor inoculation, followed by CBLB502 treatment. We monitored the survival of these mice for 80 days, and used bioluminescence imaging to measure tumor burden at various time points. CD8^+^ T cell-depleted mice exhibited higher tumor burden (Fig. 3A), and presented worse survival, with 100% lethality occurring 3–4 weeks earlier (Fig. 3B) as compared to the mice treated with PBS. In contrast, CBLB502-treated non-depleted mice exhibited lower tumor burden and significantly improved survival with the majority tumor free at the endpoint of this experiment. These results suggest that CD8^+^ T cells are important in the control of A20 tumor, and that CBLB502 treatment enhances the ability of CD8^+^ T cells to control tumor growth. Interestingly, CBLB502 treatment was able to moderately decrease the tumor burden within CD8^+^ T cell-depleted groups resulting in prolonged survival. This effect may be due to an improved recovery of CD8^+^ T cells that was evident when we examined the levels of CD8^+^ T cells post-depletion, with CBLB502-treated mice having significantly higher levels of circulating CD8^+^ T cells versus PBS controls 16 days after depletion (Fig. 3C). However, compared to PBS treatment, CBLB502 did not enhance circulating CD8^+^ T cell numbers in non-depleted mice (Fig. 3C and 2C). Interestingly, CBLB502 enhances CD8^+^ T cell recovery only in the hosts with A20 cells which express MHC I and are susceptible to CD8^+^ T cells (Fig. 3C). In contrast, CBLB502 does enhance CD8^+^ T cell recovery in the hosts with MHC I low RMAS cells (Fig. 2C).

Together, these data in Figures 2 and 3 demonstrate that CBLB502 has the ability to stimulate both innate and adaptive antitumor responses and can promote tumor clearance in multiple mouse strains and against different syngeneic tumor types.

### CD11b^+^ and CD11c^+^ cells, but not NK or T cells, Directly Respond to CBLB502 Treatment

**Figure 4:**
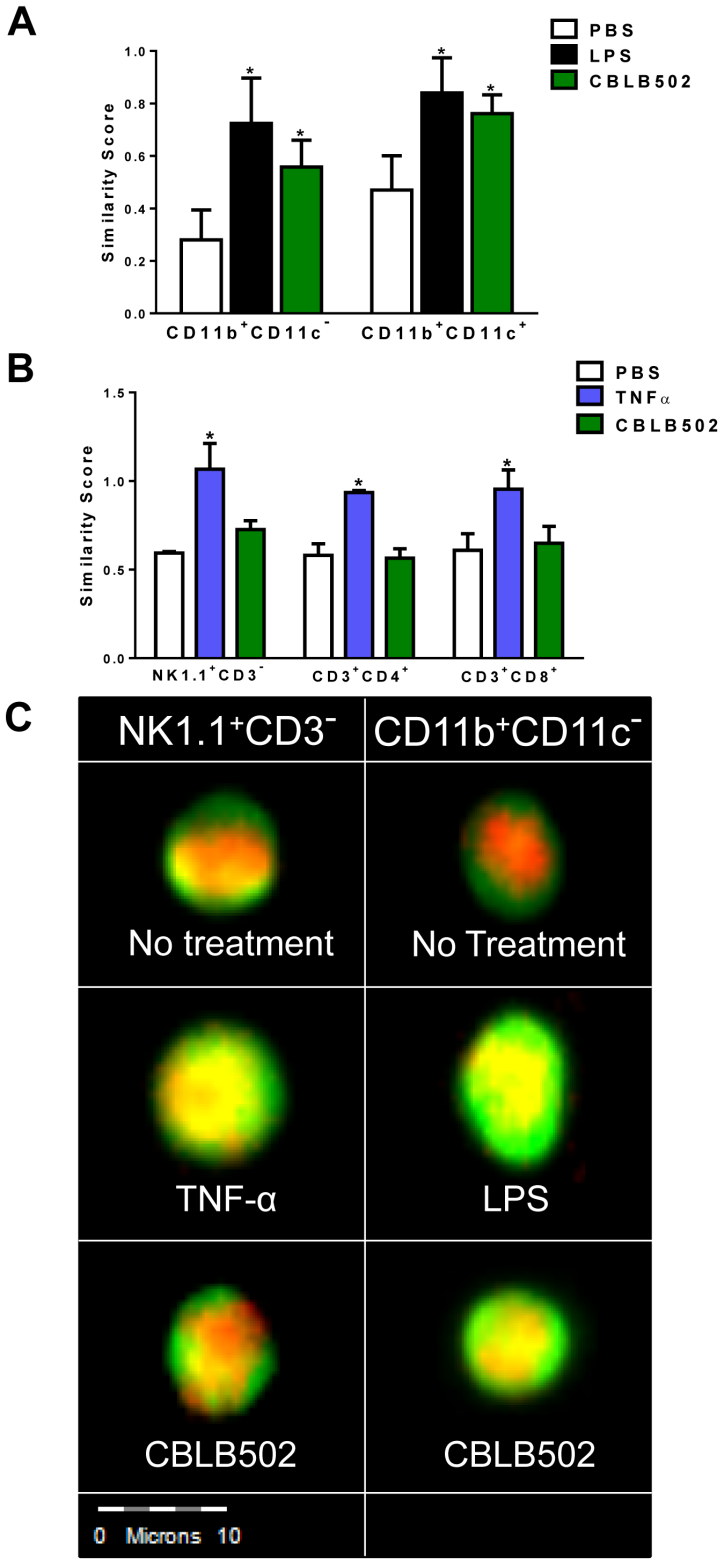
CD11b^+^ CD11c^−^ and CD11b^+^ CD11c^+^ accessory cells, but not NK or T lymphocytes, directly respond to CBLB502 treatment. Nuclear translocation of the NFκB p65 subunit was used to evaluate TLR5 expression and functionality. Splenocytes were treated with 10 ng/mL TNF-α, 100 ng/mL LPS, 100 ng/mL CBLB502 or PBS for 1 hr. (A) Splenocytes were stained for CD4, CD8, or NK1.1 along with CD3. After cell surface staining, cells were permeabilized and stained intracellularly for NFκB p65. Target populations were then gated as CD3^+^CD4^+^, CD3^+^CD8^+^ and CD3^−^NK1.1^+^ and similarity score between DAPI and the FITC-labeled NFκB was measured. (B) Splenocytes were stained for CD11b, CD11c and NFκB as described in panel A. Target populations were then gated as CD11b^+^CD11c^−^ or CD11b^+^CD11c^+^ to quantitate the similarity score. (C) Representative images at 40× magnification of NK1.1^+^CD3^−^ cells and CD11b^+^ CD11c^−^ cells treated with PBS, TNF-α, LPS, or CBLB502. Two tailed t-tests were used to determine significance versus PBS-treated control samples (*P<0.05). Data are representative from one of least three experiments.

Previous studies have largely looked at TLR5 mRNA expression due to the lack of a reliable antibody [28], [29]. However, mRNA expression does not necessarily equate to protein production or functionality and it is not fully elucidated which immune cells are in fact direct responders to flagellin. TLR5 signals through a MyD88 dependent pathway and promotes NFκB nuclear translocation, making NFκB nuclear translocation a reliable marker of TLR5 protein expression and functionality. Therefore, we used ImageStream cytometry to monitor NFκB nuclear translocation to identify the cells that directly respond to CBLB502. NFκB nuclear translocation was quantified via similarity score, which measures the co-localization between the nuclear dye DAPI (red) and the FITC-labeled NFκB p65 subunit (green). The similarity score becomes high (yellow) when there is co-localization between DAPI (red) and NFκB-FITC (green). Thus, as the amount of nuclear NFκB increases so does similarity score. Cells in which NFκB is mainly cytoplasmic after treatment will have a low similarity score and will be considered to non-responders to TLR5 agonists. To determine the ability of cells to respond to CBLB502, splenocytes were treated with LPS or TNF-α as positive controls, or treated with CBLB502 or PBS for 1 hour. One hour was used to establish which cells were direct responders to CBLB502 and ideally eliminate any secondary responders showing NFκB nuclear translocation. As shown in Figure 4A, TNF-α treatment significantly increased the similarity score of the NK1.1^+^CD3^−^ NK cells, CD3^+^CD4^+^ and CD3^+^CD8^+^ T cells. In contrast, the similarity score of these NK and T cells remains unchanged when comparing PBS and CBLB502 treatments, indicating that these lymphocytes do not respond to CBLB502 within 1 hr of treatment *in vitro*. These results suggest that some accessory cell types may directly respond to CBLB502 and subsequently activate NK and T cells.

Based on this hypothesis, we examined the similarity score of CD11b^+^ and CD11c^+^ cells. As shown in Figure 4B, both splenic CD11b^+^CD11c^−^ and CD11b^+^CD11c^+^ cells responded to CBLB502 or LPS treatment as indicated by NFκB nuclear translocation. Representative images of NFκB nuclear translocation in NK and CD11b^+^ CD11c^−^ cells are shown in Figure 4C. As both CD11b^+^ and CD11c^+^ cells are capable of antigen presentation and production of pro-inflammatory cytokines that can drive cytotoxic immune responses, it is likely that these cells are the bridge between TLR5-mediated stimulation and cytotoxic eradication of tumor cells.

### Up-regulation of Co-stimulatory Molecules and Pro-inflammatory Cytokines after CBLB502 Treatment

**Figure 5:**
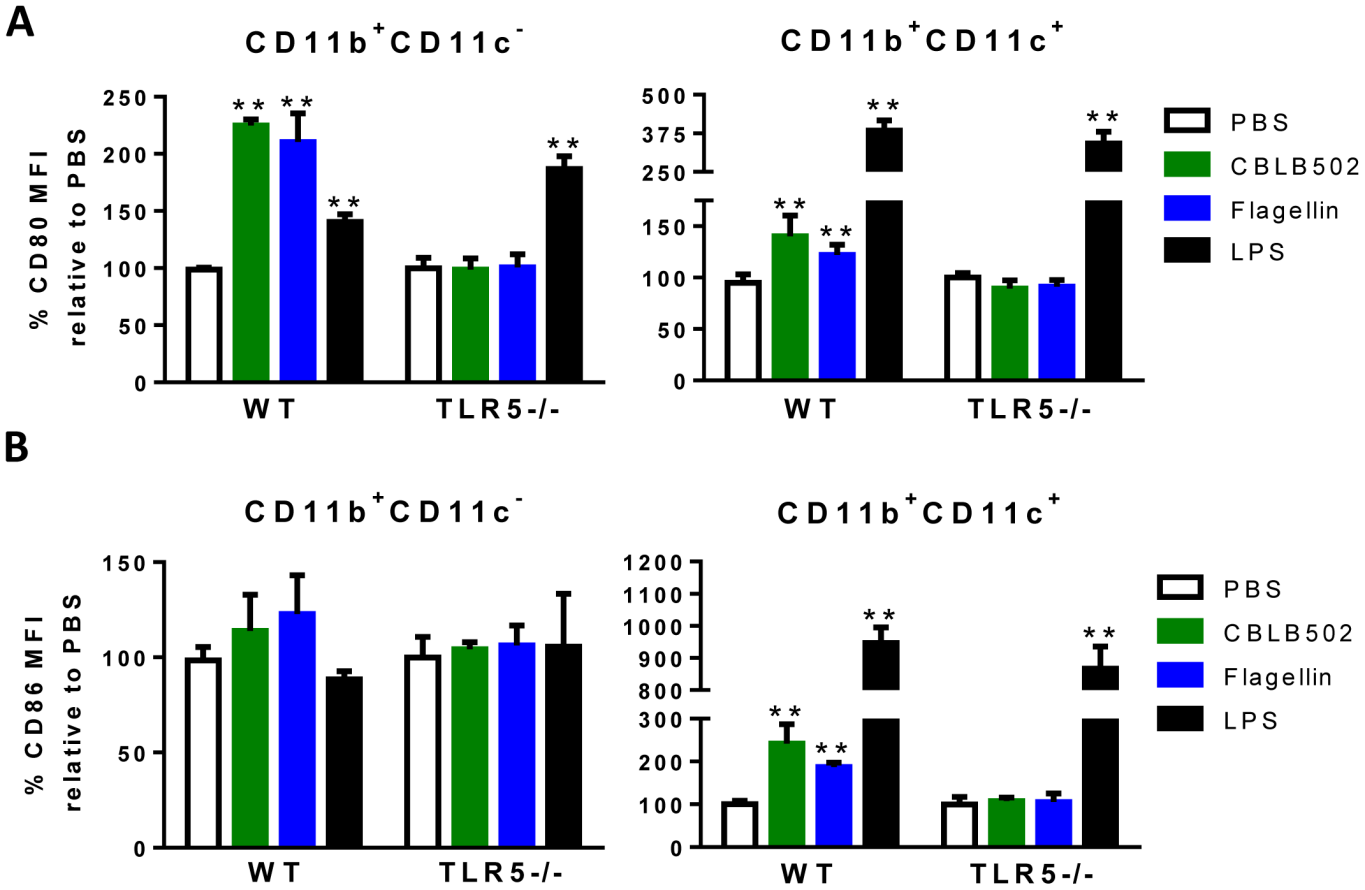
CD80 and CD86 are up-regulated *in vivo* after CBLB502 treatment in a TLR5 dependent manner. WT or TLR5^−/−^ C57BL/6 mice were injected s.c. with 1 µg CBLB502, 1 µg flagellin, or equivalent volume of PBS. 24 h post-injection splenocytes were harvested and stained for flow cytometry with CD11b, CD11c, CD80 and CD86 antibodies. For LPS injections, 10 µg LPS was injected intraperitoneally and splenocytes were harvested 6 hours later and stained with the aforementioned antibodies immediately. (A) CD80 expression on CD11b^+^CD11c^−^ and CD11b^+^CD11c^+^ cells from WT and TLR5^−/−^ mice after indicated treatments. (B) CD86 expression on these same cell subsets. Two tailed t-tests were used to determine significance versus PBS-treated controls (**P<0.01, n = 3–6 mice per group). Data are representative from one of least three experiments.

Since both CD11b^+^ and CD11c^+^ are direct responders to CBLB502 and these same cell types are capable of antigen presentation, providing co-stimulation, and producing pro-inflammatory cytokines, we focused on the response of these cells to CBLB502 treatment. Previous reports suggest that flagellin has the ability to up-regulate co-stimulatory molecules and increase pro-inflammatory cytokines [8], [30]. To address whether CBLB502 is enhancing immunity via up-regulation of co-stimulatory molecules, we treated mice with PBS, CBLB502, LPS or full-length flagellin and examined direct responders to CBLB502 (e.g. CD11b^+^ and CD11c^+^). Treatment with CBLB502, flagellin or LPS all caused significant up-regulation of CD80 on CD11b^+^CD11c^−^ and CD11b^+^CD11c^+^ cells in WT mice (Fig. 5A). However, when TLR5^−/−^ mice were given the same treatments only LPS caused an up-regulation of CD80 (Fig. 5A). This suggests that the stimulating effect of CBLB502 is indeed through TLR5 and that contaminating endotoxin is not contributing to CD80 up-regulation. All three treatments also caused an up-regulation of CD86 on CD11b^+^CD11c^+^ in WT mice, however, none of the treatments up-regulated CD86 expression on CD11b^+^CD11c^−^ cells (Fig. 5B). Other groups have reported that CD80 and CD86 do not necessarily up- or down-regulate in parallel, a result that is consistent with the CD80 and CD86 expression on CD11b^+^CD11c^−^ cells in this model [31], [32]. In TLR5^−/−^ mice, LPS was again the only treatment that caused up-regulation of CD86 on CD11b^+^CD11c^+^ (Fig. 5B). This suggests that these accessory cells up-regulate co-stimulatory molecules in response to CBLB502 in a TLR5 dependent manner. As co-stimulation is an important aspect of productive T cell responses, this up-regulation after treatment may contribute to the effects of CBLB502 on antitumor immunity.

To assess the ability of CBLB502 to increase pro-inflammatory cytokines we evaluated a selection of cytokines associated with NK and T cell activation. As seen in Table 1, in response to CBLB502 treatment, IFNγ, IL-6, IL-15, IL-12p70 and IL-12p40 were up-regulated. Notably, IL-15 is a potent activator of NK cell cytotoxicity [16]. In addition, increases in IFNγ, IL-6, and IL-12 may contribute to the T cell activation and subsequent antitumor response we observed in mice challenged with A20 tumor [33], [34]. The marked increase in these pro-inflammatory cytokines coupled with the up-regulation of co-stimulatory molecules on accessory immune cells supports the hypothesis that CBLB502 is providing a favorable environment for a productive antitumor response.

**Table 1:** Plasma levels of pro-inflammatory cytokines are increased by CBLB502 treatment.

C57BL/6	IFNg	IL-6	IL-7	IL-12p40	IL-12p70	IL-15
**Unstimulated**	0	7.355	1.195	20.005	15.645	40.255
**30 min**	20.89	425.42	14.585	16.9	29.57	84.01
**2 h**	150.33	7355.29	31.69	199.775	141.175	122.295
**4 h**	73.36	743.32	21.9	97.16	99.59	113.18

C57BL/6 mice were treated s.c. with 5 µg CBLB502 and blood was collected via cardiac puncture from two mice per time point. Values depict concentration in plasma in pg/mL of various cytokines.

## Discussion

Various TLR agonists have been tested in a wide array of tumor models and clinical trials [35]. Multiple mechanisms of action have been described including inducing apoptosis [36], altering proliferation [13], and promoting an immune response [11], [37]. Since tumors are host-derived cells, it is inherently difficult for the immune system to recognize them as immunogenic. An important component of a productive immune response is the recognition of antigen in the presence of a danger signal. Based on this rationale, the TLR5 agonist CBLB502 was administered and shown to promote tumor clearance in C57BL/6 and BALB/c mice. In this study, we utilize TLR5-negative tumors ruling out direct killing or alteration in proliferation of the tumors by CBLB502, leaving the host as the sole mediator of tumor clearance. The addition of CBLB502 adds the integral danger context, with accessory cells responding to CBLB502 and subsequently increasing tumor clearance by both NK and CD8^+^ T cells.

These accessory cell types have been documented to promote NK cell proliferation upon flagellin administration [38], which is consistent with our finding that CBLB502 directly stimulated CD11c^+^ cells and enhanced NK cell-dependent tumor clearance. What has not been clear up to this point is whether splenic dendritic cells have the ability to respond to TLR5-meidatied stimulus, with some studies suggesting that they are direct responders and others that they are activated as bystanders [28], [39], [40]. With the use of ImageStream, we show here that splenic CD11c^+^ cells are directly responsive to TLR5 agonists. This makes CD11b^+^ and CD11c^+^ cells central to the activity observed after CBLB502 administration.

As for prior tumor studies using flagellin, administration of flagellin alone was shown to slow tumor growth in a Her-2 transfected tumor line, but not its non-antigenically modified parental line [11]. This same study showed that flagellin administration starting day 0 promoted tumor growth; however, these studies used a subcutaneous tumor model. Flagellin could be promoting tumor initiation for solid subcutaneous tumors, or location of the tumor may be a factor in the efficacy of flagellin treatment. In fact, a recent report suggests that the liver is a primary responder to TLR5 agonists and after treatment both NK and T cells are recruited to the liver. Interestingly, the liver is an organ in which A20 lymphomas have a tropism [41], [42].

Modifications to flagellin have also proven useful, both flagellin derivatives/antigen delivered via liposomes [43] and flagellin fusions have shown efficacy at inducing immune response to tumors [44]. However, unmodified flagellin does not provide potent immune stimulatory activity in conjunction with antigen. That is, vaccination before tumor inoculation with recombinant flagellin administered with recombinant OVA could not provide a protective effect that was seen with OVA-flagellin fusions [44]. This lack of protection may be due to the fact that flagellin in combination with antigen was not as efficacious as flagellin fused with antigen in generating an antigen-specific CD8^+^ T cell response, though interestingly this was independent of conventional TLR5 signaling [45]. Further work with CBLB502 will reveal whether it has similar characteristics to flagellin in terms of mediating CD8^+^ T cell responses.

As future studies will further elucidate the role of TLR5 in antitumor immunity, we do not anticipate any fundamental difference between CBLB502 and flagellin with regard to the mechanism of action. In fact, Figure 5 and Table 1 are evidence that CBLB502 has similar activity to flagellin, as co-stimulation up-regulation and cytokine production after CBLB502 treatment are similar to that of flagellin treated mice [8], [30], [39], [40]. However, comparative studies have shown that CBLB502 is less toxic than flagellin and induces a lower neutralizing antibody response [14]. As a result, a Phase I clinical trial to evaluate the effect of CBLB502 on solid tumors that are unable to be removed by surgery is currently ongoing.

To use CBLB502 clinically it will be essential to evaluate whether an individual has functional TLR5, as reports suggest that up to 10% of individuals may have an impaired ability to respond to flagellin due to a stop codon mutation in the *TLR5* gene [46]. This makes functional readouts, such as NFκB nuclear translocation, a necessity in determining TLR5 status. We implement a technique using ImageStream flow cytometry that allows for evaluation of primary cell TLR5 status without the need for a TLR5 specific antibody. This same technique can be used to evaluate functionality of any TLR signaling, which will be of significance going forward with the use of new TLR-based adjuvants.

In line with the adjuvant notion, our study shows that CBLB502 is a promising adjuvant as it induces potent antitumor responses in two syngeneic lymphoma models without targeting known tumor antigens. Going forward, a practical strategy should focus on harnessing the danger context induced by TLR5 stimulation. Use of a TLR agonist such as CBLB502 to alter the tumor microenvironment, combined with promoting innate and adaptive responses to defined tumor-specific antigens, should enhance the efficacy of vaccine-based tumor immunotherapy.
